# Relationship between sleep disorders and depression in perimenopausal women in the United States: a cross-sectional survey based on NHANES 2009-2014

**DOI:** 10.4314/ahs.v25i1.40

**Published:** 2025-03

**Authors:** Le Zhang, Hao Wang, Yuanqing Shen, Fangyao Xie, Miaomiao Xu, Bing Xiong

**Affiliations:** Department of TCM Rehabilitation, The second Affiliated Hospital of Zhejiang University School of Medicine, Hangzhou, China

**Keywords:** sleep disorders, depression, perimenopausal women, NHANES

## Abstract

**Background:**

Many studies have found that depression and sleep disorders in perimenopausal women often appear as comorbidities, but its relevance has not been further verified. To clarify the relationship between the two can provide guidance for future clinical research.

**Methodology:**

A cross-sectional research was carried out among 1400 women aged 45-55 years who participated in the 2009-2014 national health and Nutrition Examination Survey (NHANES). We estimated unadjusted and adjusted logistic regression models to analyze the relationship between sleep disturbances and other related factors with depression.

**Results:**

18.1% of the 1440 participants had depressive symptoms in the past two weeks. 11.2% had sleep disorder. Compared with women without sleep disorder (14.70%), depression was more common in perimenopausal women who had sleep disorder (44.72%), In the logistic regression model, menopausal women with sleep disturbance suffer from depression were four times more than that without sleep disturbance (odds ratio, 3.798; 95%CI, 2.611-5.525); The difference remained significant after adjusted the effects of covariates (adjusted odds ratio, 3.687; 95% CI, 2.533-5.367).

**Conclusion:**

Sleep disorders strongly link to depression in menopausal women. Early intervention for sleep issues may reduce depression risk in this group.

## Introduction

Perimenopause is considered to be the most common and critical stage in the development of depression in women's life cycle. At this stage, the prevalence of depression is particularly high^1^. Perimenopausal depression is characterized by emotional disorders and menopausal specific physical symptoms. Some studies have shown that the quality of life, social support and fitness of depressed women is significantly reduced compared to healthy women, and the disability is increased, leading to the decline of their socil skills and quality of life^2^. Guidelines for the assessment of this kind of women were developed in 2018 by the NAMS trustees board and the national depression Center Network Working Group on women and mood disorders. It is pointed out that sleep disorder is a common accompanying symptom in middle-aged and elderly depression^3^. A number of studies have pointed out that women are more at risk of sleep disorderthan men because of reproductive hormone changes, stress, depression, aging, life role changes and other factors, and their sleep quality is generally poor^4^. Sleep disorders are also the most common disease in perimenopausal women. In this period of dramatic changes in reproductive hormones, women's risk of insomnia will increase significantly^5^. Although many studies have found that depression and sleep disorders in perimenopausal women often appear as comorbidities^6^,^7^ its relevance has not been further verified. There have been studies suggesting that insomnia in menopausal and postmenopausal women in Japan is closely related to depression^8^. But such research is still blank in American Menopause Women. Therefore, we aimed to design a study that could represent the menopausal female population in the United States, with a large sample size, controlled for confounders, and included reliable measures of depression and sleep disorder. We found the NHANES database, which is the basis for nutrition surveillance to inform nutrition policy. Since the early 1970s, NHANES has conducted a nationwide representative sampling survey and collected detailed data on daily diet and major chronic diseases^9^. This database meets the needs of this study.

## Methods

### Data Sources

NHANES is a large-scale, continuous, and representative cross-sectional survey. Data are freely available to researchers by accessing the NHANES website^9^. A cross-sectional research was carried out on 1684 women aged 45-55 years and participated NHANES during 2009-2014. We excluded 234 participants with missing outcome variable depression, 4 participants with missing sleep disorder data and 4 participants with missing important covariates. Finally, the remaining 1400 women were included for the study. All participants provided informed consent.

### Outcome

Depressive symptoms as a dependent variable in this study. Thedepression condition of the participant were tested based on the patient health questionnaire (PHQ-9), which is a nine-item screening tool related to the frequency of depressive symptoms recently. Inme-ta-analysis, investigators found that the PHQ-9 was more sensitive than a previous conventional meta-analysis combining reference standards when compared with a semi-structured diagnostic interview^10^. A final follow-up question assessed the overall impairment situation. If the PHQ-9 score was greater than 9, the whole participants were divided as having depressive symptoms, and we used this threshold. In a recent systematic evaluation of PHQ-9 for depression screening, it was concluded that the overall sensitivity of PHQ-9 was 0.37 to 0.98, and the specificity was 0.42 to 0.99. This shows that PHQ-9 has been widely validated and recommended for the two-stage screening process^11^.

### Exposure

Sleep disorders was used as an independent variable in this study. A computer-assisted personal interview (CAPI) system was used to ask about sleep disturbances. All of the research object were asked the questions about sleep difficulties: “have you ever told a doctor that you have sleep difficulties?” If they responded positively to this question, it can be inferred that, they have sleeping disorders^12^. Therefore, the clinical diagnosis of professionals is taken as the standard to judge whether the participants have sleep disorders.

### Descriptive variables

Descriptive variable includes the age group of menopausal women (45-50,50-55), race(Mexican American, Other Hispanic, Non-Hispanic White, Non-Hispanic Black, Other Race), education level(Less than 9th grade, 9-11th grade, GED or equivalent, Some university graduate or above). The ratio of family income to poverty (0-4.99, Value more than or equal to 5.00, missing), high blood pressure (yes, high vs no, does not have), high cholesterol level (yes, has high cholesterol level vs no, does not have high cholesterol level), diabetes (yes, has diabetes vs no, has not), health insurance (yes, has health insurance vs no, have not health insurance). All the data above were obtained in NHANES database.

### Statistical analysis

Data analysis for this study considered sampling weights according to the analysis guidelines edited by the NCHS. In this research, data were analyzed using the software package r and empower software. A descriptive analysis of the research population was conducted.in this research, we used χ^2^ analysis to perform bivariate analysis between variable (sleep disturbance) and depression. Considering that depression is a binary variable in the research process, the adjusted and unadjusted models were selected in the regression analysis process, and the correlation between other factors and sleep disorders was determined on this basis. When processing the regression results of all independent variables, the odds ratio (or) and 95% confidence interval (CI) were set and the results were compared. The significance level was 0.05.

## Results

### Prevalence and Demographic Characteristics of Participants

Of the 1440 individuals, 260 (18.1%) showed depressive symptoms in the previous two weeks (see Table 1). 161 cases (11.2%) had sleep disorder. There were 793 participants aged 45-50 and 657 cases aged 51-55. There exists no obvious difference between the two age groups. Most of them were non Hispanic white (42.0%), followed by non Hispanic black (23.4%), Mexican American (13.5%), other Hispanic (11.3%) and other race (9.7%). 29.9% of them was high school graduate / GED or equivalent, and 26.6% of women graduated from university. Among them, 59.9% of the female married / livingith partner, and most of them (72.1%) were in relatively low ratio of family income to poverty (range of values 0-4.99). Among these menopausal women, some have chronic diseases, of which 37.3% have hypertension, 36.3% have hyperlipidemia, and 11.9% have diabetes. 76.5% of the participants had medical insurance.

**Table 1:** Weighted Characteristics of Study Sample (N = 1,440), Women Aged 45-55

	Trouble sleeping		Total,n(%)

	No,n(%)	Yes,n(%)	
	1279(88.8%)	161(11.2%)	1440(100.0)
depression in previous 2weeks			
no	1091 (85.3%)	89 (55.3%)	1180(81.9%)
yes	188 (14.7%)	72 (44.7%)	260(18.1%)
age,y^a^			
45-50	708 (55.4%)	85 (52.8%)	793(55.0%)
51-55	571 (44.6%)	76 (47.2%)	647(45.0%)
Race			
Mexican American	185 (14.5%)	10 (6.2%)	195(13.5%)
Other Hispanic	141 (11.0%)	22 (13.7%)	163(11.3%)
Non-Hispanic White	522 (40.8%)	83 (51.6%)	605(42.0%)
Non-Hispanic Black	302 (23.6%)	35 (21.7%)	337(23.4%)
Other Race	129 (10.1%)	11 (6.8%)	140(9.7%)
education level			
Less than 9th grade	307 (24.0%)	32 (19.9%)	339(23.5%)
9-11th grade	251 (19.6%)	37 (23.0%)	288(20.0%)
High school graduate/GED or equivalent	372 (29.1%)	58 (36.0%)	430(29.9%)
Some college graduate or above	349 (27.3%)	34 (21.1%)	383(26.6%)
marital status			
Married/Living with partner	791 (61.8%)	71 (44.1%)	862(59.9%)
Widowed/Divorced/Separated	327 (25.6%)	66 (41.0%)	393(27.3%)
Never married	161 (12.6%)	24 (14.9%)	185(12.8%)
Ratio of family income to poverty^b^			
Range of Values 0-4.99	908 (71.0%)	130 (80.7%)	1038(72.1%)
Value greater than or equal to 5.00	260 (20.3%)	21 (13.0%)	281(19.5%)
missing	111 (8.7%)	10 (6.2%)	121(8.4%)
has high blood pressure			
yes	452 (35.3%)	85 (52.8%)	537(37.3%)
no	827 (64.7%)	76 (47.2%)	903(62.7%)
has high cholesterol level			
yes	448 (35.0%)	74 (46.0%)	522(36.3%)
no	733 (57.3%)	78 (48.4%)	811(56.3%)
missing	98 (7.7%)	9 (5.6%)	107(7.4%)
has diabetes			
yes	140 (10.9%)	31 (19.3%)	171(11.9%)
no	1105 (86.4%)	120 (74.5%)	1225(85.1%)
missing	34 (2.7%)	10 (6.2%)	44(3.0%)
has health insurance			
yes	968 (75.7%)	134 (83.2%)	1102(76.5%)
no	311 (24.3%)	27 (16.8%)	338(23.5)
Abbreviation: SD, standard deviation.			
Percentages and means were weighted and incorporated NHANES sample weights.			

a. Mean (SD) age was 49.9(2.9) years.

b. Mean (SD) Ratio of family income to poverty was 2.7(1.7).

### Associations Between Depression and Sleep Disorders, Education Level, Marital Status, and Socioeconomic Factors

Compared with women without sleep disorder (14.70%), depression was more common in perimenopausal women who had sleep disorder (44.72%), and it was more common in women with education level of less than 9th grade (25.66%), followed by high school graduate / GED or equivalent (21.4%), 9-11th grade (18.06%) and Some college graduate or above (7.57%). The female population of widowed / divorced / separated was more likely to suffer from depression (26.72%), followed by never married (22.70%). Married / living with partner (13.11%) was the rarest in female population. The proportion of women suffering from depression in the family with ratio of family income to poverty (21.00%) was significantly higher than that in the family with value greater than or equal to 5.0 (6.76%). The proportion of women with hypertension (24.77%) was significantly higher than that without hypertension (14.06%). Similarly, the incidence of depression was significant higher with high cholesterol level (22.99% vs 14.92%), with diabetes than without diabetes (28.65% vs 16.24%). The trend was not significant in age, race, and Medicare. In the data analysis, we found a strong correlation between depression and sleep disorders. In unadjusted logistic regression model, menopausal women with depression and sleep disorders were more than 3 times higher than without sleep disorders (OR, 3.798; 95%CI,2.611-5.525); After adjusting for covariate factors, the risk of depression in menopausal women with sleep disorders was still significantly higher than that without sleep disorders (AOR, 3.687; 95%CI,2.533-5.367). At the same time, we found that the education level is inversely correlated to depression. For the adjusted regression model, women with some group graduate or above had the lowest risk of depression, and less than 9th grade had more than three times the risk of depression of college graduates (AOR, 3.603; 95%CI, 2.195-5.913) (Table 2).

**Table 2:** Relationship Between Trouble Sleeping and Depression among (N = 1,440) Women Aged 45-55 NHANES, 2009–2014

Variable	Depression in Previous 2 Weeks		Total	*χ*^2^ test	P value
Depression in previous 2weeks					
no	1091 (85.30%)	188 (14.70%)	1279 (100%)	87.1	<0.01
yes	89 (55.28%)	72 (44.72%)	161 (100%)		
AGE					
45-50	654 (82.47%)	139 (17.53%)	793 (100%)	0.3	0.56
51-55	526 (81.30%)	121 (18.70%)	647 (100%)		
RACE					
Mexican American	167 (85.64%)	28 (14.36%)	195 (100%)		
Other Hispanic	130 (79.75%)	33 (20.25%)	163 (100%)		
Non-Hispanic White	482 (79.67%)	123 (20.33%)	605 (100%)	6.6	0.16
Non-Hispanic Black	280 (83.09%)	57 (16.91%)	337 (100%)		
Other Race	121 (86.43%)	19 (13.57%)	140 (100%)		
education level					
Less than 9th grade	252 (74.34%)	87 (25.66%)	339 (100%)		
9-11th grade	236 (81.94%)	52 (18.06%)	288 (100%)		
High school graduate/GED or equivalent	338 (78.60%)	92 (21.40%)	430 (100%)	44.9	<0.01
Some college graduate or above	354 (92.43%)	29 (7.57%)	383 (100%)		
marital status					
Married/Living with partner	749 (86.89%)	113 (13.11%)	862 (100%)		
Widowed/Divorced/Separated	288 (73.28%)	105 (26.72%)	393 (100%)	36.9	<0.01
Never married	143 (77.30%)	42 (22.70%)	185 (100%)		
Ratio of family income to poverty					
Range of Values 0-4.99	820 (79.00%)	218 (21.00%)	1038 (100%)		
Value greater than or equal to 5.00	262 (93.24%)	19 (6.76%)	281 (100%)	30.39	<0.01
missing	98 (80.99%)	23 (19.01%)	121 (100%)		
had high blood pressure					
yes	404 (75.23%)	133 (24.77%)	537 (100%)	26.1	<0.01
no	776 (85.94%)	127 (14.06%)	903 (100%)		
high cholesterol level					
yes	402 (77.01%)	120 (22.99%)	522 (100%)		
no	690 (85.08%)	121 (14.92%)	811 (100%)	13.9	<0.01
missing	88 (82.24%)	19 (17.76%)	107 (100%)		
	has diabetes				
yes	122 (71.35%)	49 (28.65%)	171 (100%)		
no	1026 (83.76%)	199 (16.24%)	1225 (100%)	18.2	<0.01
missing	32 (72.73%)	12 (27.27%)	44 (100%)		
	has health insurance				
yes	906 (82.21%)	196 (17.79%)	1102 (100%)	0.23	0.63
no	274 (81.07%)	64 (18.93%)	338 (100%)		

a Percentages are row percentages.

### Association Between Marital Status, Socioeconomic Factors, Chronic Diseases, and Depression Risk

In the adjusted regression model, we found that the risk of depression of married / living with partner women was significantly lower than that of single women (AOR, 0.516; 95%CI, 0.335-0.795). Among them, ratio of family income to poverty was inversely proportional to the risk of depression. The group with higher ratio of family income to poverty had lower risk of depression (AOR, 0.387; 95%CI, 0.196-0.766). In observing the relationship between common chronic diseases and depression in middle-aged and elderly people, women with hypertension were obviously more prone to depression (AOR, 1.429; 95%CI, 1.048-1.947); Women with hyperlipidemia and diabetes had no significant risk of depression. There is no direct relation between medical insurance and the risk of depression (Table 3).

**Table 3:** Odds Ratios and Confidence Intervals for the Logistic Regression Estimate for the Relationship Between Trouble Sleeping and Depression Among Women Aged 45–55

Covariate	Unadjusted Model		Adjusted Model	

	OR(95% CI)	P Value	AOR(95% CI)	P Value
depression in previous 2weeks				
yes	3.798(2.611-5.525)	<.001	3.687(2.533-5.367)	<.001
no	1 [Reference]		1 [Reference]	
age				
51-55	1 [Reference]		1 [Reference]	
45-50	1.073(0.796-1.447)	0.644	1.039(0.774-1.395)	0.799
Race				
Mexican American	0.601(0.299-1.205)	0.151	0.526(0.272-1.014)	0.055
Other Hispanic	0.943(0.481-1.850)	0.865	0.822(0.437-1.546)	0.542
Non-Hispanic White	1.250(0.708-2.207)	0.442	1.109(0.655-1.876)	0.701
Non-Hispanic Black	0.706(0.381-1.311)	0.271	0.616(0.348-1.093)	0.098
Other Race	1 [Reference]		1 [Reference]	
education level				
Less than 9th grade	3.940(2.347-6.615)	<.001	3.603(2.195-5.913)	<.001
9-11th grade	2.004(1.189-3.378)	0.009	1.843(1.116-3.044)	0.017
High school graduate/GED or equivalent	2.440(1.510-3.944)	<.001	2.232(1.415-3.521)	0.001
Some college graduate or above	1 [Reference]		1 [Reference]	
marital status				
Married/Living with partner	0.550(0.352-0.859)	0.009	0.516(0.335-0.795)	0.003
Widowed/Divorced/Separated	1.026(0.651-1.616)	0.914	0.969(0.622-1.509)	0.888
Never married	1 [Reference]		1 [Reference]	
Ratio of family income to poverty				
Range of Values 0-4.99	0.923(0.552-1.543)	0.760	0.826(0.513-1.329)	0.430
Value greater than or equal to 5.00	0.435(0.213-0.886)	0.022	0.387(0.196-0.766)	0.006
missing	1 [Reference]		1 [Reference]	
has high blood pressure				
yes	1.452(1.065-1.980)	0.019	1.429(1.048-1.947)	0.024
no	1 [Reference]		1 [Reference]	
has high cholesterol level				
yes	1.365(0.754-2.469)	0.304	1.186(0.690-2.039)	0.536
no	0.994(0.562-1.760)	0.985	0.862(0.514-1.443)	0.571
missing	1 [Reference]		1 [Reference]	
has diabetes				
yes	1.049(0.457-2.408)	0.909	0.809(0.395-1.658)	0.563
no	0.812(0.377-1.749)	0.595	0.622(0.329-1.175)	0.144
missing	1 [Reference]		1 [Reference]	
has health insurance				
yes	1.062(0.747-1.511)	0.737	1.007(0.715-1.417)	0.97
no	1 [Reference]		1 [Reference]	

Abbreviations: AOR, adjusted odds ratio; CI, confidence interval; OR, odds ratio.

a P values were determined by t test.

## Discussion

Sleep disorder and depression are common problems in menopausal women, but there are few studies on the relationship between them and their interaction is still unclear. Numerous longitudinal studies have defined sleep disturbance as a risk factor for new or recurrent depression at various ages. An interesting point is derived here: sleep problems is a predictable precursor^13^. The same conclusion has been found in other studies: Sleep disturbances were closely associated with later depression progression in adults compared to older age groups^14^,^15^. In this study, we designed a research so as to evaluate the relation of that in perimenopausal women, whose findings is representative of the American population. We found that there was an obvious correlation between sleep disorders and depression in this population. After adjusting for relevant factors such as socioeconomic, demographic and common chronic diseases (race, age, education level, marginal status, population status, health insurance status, diabetes, high blood pressure), the association remained significant. Nowadays these kind of relation in perimenopausal women has not been fully revealed, several meta-analyses corroborate our findings that sleep disturbances such is very common in the menopausal transition, with an approximate prevalence of 8.4-56.6% for menopause related sleep disorders^16^. The incidence rate of sleep disorders is also in this range. At the same time, it can be inferred that education level is negative correlated to the incidence of depression in menopausal women. For example, women who graduated from college or above had the lowest prevalence of depression. The AOR of below 9th, 9-11th grade graduate were 3.603, 1.843 and 2.232 respectively, there were significant statistical significance. In other studies, we found that young women (20-30y) with lower education level are prone to suffer from depression than higher education level women^12^. Related studies also found that during the transition period of menopause, due to the duration of vasomotor symptoms, the degree of depression and anxiety is higher, and other factors include lower education level, higher perceived stress and symptom sensitivity^17^. The above studies have found that women and low education level are important factors of depression, which is in accordance with the conclusion of this research. Therefore, although the mechanism is not clear, we propose that higher education level has a protective effect on depression in menopausal women. We also discovered a correlation between depression and marital status among our study participants. Compared to single and never-married women, those who were married or living with a partner had a significantly lower risk of experiencing depression. These findings are supported by a previous study that examined depression in the elderly, which found that women, widows, and single individuals were more likely to be associated with depression^18^. Another cohort study examined this association and suggested that women, people living alone, and those with lower education levels were prone to developing depression. Therefore, it is apparent that menopausal women who live alone and lack a partner may exhibit higher sensitivity to emotions, lower stress tolerance, and an increased risk of depression. However, since our study specifically focused on menopausal women, it is possible that women in this age group are more susceptible to loneliness, making the influence of marital status biased. Consequently, further research is necessary to establish a more precise argument. The result of this paper implied that the risk of depression is closely related to the economic status of menopausal women's families: the higher the ratio of family income, the lower the risk of depression. A recent population-based study in a rural area of Sichuan Province, China, found that annual family income per capita was significantly associated with depression. The study called for more attention to women and low-income groups^19^. Many relevant research had found that low income was strong related to female depression^20^,^21^. Therefore, reasonable measures to improve the family income of menopausal women will play a positive role in reducing depression risk. At the same time, we found that the relation between hypertension and depression in representative chronic diseases is very obvious. Menopausal women with hypertension have a higher risk of depression. In a research examining the relation of major depressive disorder and arterial hypertension in a Colombian population, depression was assigned as a risk factor for hypertension, while 22.2% of patients with hypertension were later diagnosed with depression, and there was a two-way risk relationship between depression and hypertension^22^. Prevention and control of hypertension will be an important target to reduce menopausal depression risk in the future. Although χ^2^ test exhibits that women with and diabetes had a significantly risk of depression than those who did not suffer from chronic diseases, their OR and AOR values were not statistically significant in logistic regression analysis.

We analyzed the reasons, which may be related to the small sample size, a small part of the missing data and other uncontrollable factors. A cross-sectional NHANES study found: compared with higher levels, low levels of total cholesterol is related to depression with lower risk^23^. But the increased risk was not associated wth low cholesterol levels^23^. In a cohort study, researchers found that regardless of demographic status, complications or drug compliance, the incidence rate of depression raised with the progression of diabetic difficult, suggesting that depression risk is associated with a variety of DM related complications and severe progression progression rates^24^. All these suggest that the occurrence and development of chronic diseases can increase the risk of depression, but its association in menopausal women is worthy of further study. Our research has several advantages. First of all, our data is obtained from a large sample and a American population database, and we conducted a logistic regression analysis on the data, calculated OR value and AOR value, and reasonably elaborated the correlation between related variables and depression. However, Our study still has the following limitations: firstly, the sample size of menopausal women is relatively small, which may cause bias in data analysis; Secondly, a small amount of data loss in some covariates may cause bias to the results; Furthermore, in the definition of depression, PHQ-9 provided by the database is the main diagnostic criteria for depression. Although many literatures point out that PHQ-9 is a simple, fast, tool for evaluating the severity of depression^25^, and the depression in this research was not rigid diagnosed, which may lead to the problem of low accuracy.

## Conclusion

We found that depression was strongly related to sleep disorders in menopausal women. This suggests that sleep disorder may be an important risk factor for menopausal women to have depression. Early intervention of sleep disorder may reduce the risk of depression in women of this special age.
